# Aux/IAA14 Regulates microRNA-Mediated Cold Stress Response in *Arabidopsis* Roots

**DOI:** 10.3390/ijms21228441

**Published:** 2020-11-10

**Authors:** Mohammad Aslam, Kenji Sugita, Yuan Qin, Abidur Rahman

**Affiliations:** 1 Department of Plant Bio Sciences, Faculty of Agriculture, Iwate University, Morioka 020-8550, Japan; aslampmb1@gmail.com (M.A.); g0419022@iwate-u.ac.jp (K.S.); 2 State Key Laboratory for Conservation and Utilization of Subtropical Agro-Bioresources, Guangxi Key Lab of Sugarcane Biology, College of Agriculture, Guangxi University, Nanning 530004, China; yuanqin@fafu.edu.cn; 3 Key Laboratory of Genetics, Breeding and Multiple Utilization of Crops, Ministry of Education, Fujian Provincial Key Laboratory of Haixia Applied Plant Systems Biology, Center for Genomics and Biotechnology, College of Agriculture, Fujian Agriculture and Forestry University, Fuzhou 350002, China; 4 United Graduate School of Agricultural Sciences, Iwate University, Morioka 020-8550, Japan; 5 Agri-Innovation Center, Faculty of Agriculture, Iwate University, Morioka 020-8550, Japan

**Keywords:** cold stress, auxin, microRNA, *Aux/IAA*

## Abstract

The phytohormone auxin and microRNA-mediated regulation of gene expressions are key regulators of plant growth and development at both optimal and under low-temperature stress conditions. However, the mechanistic link between microRNA and auxin in regulating plant cold stress response remains elusive. To better understand the role of microRNA (miR) in the crosstalk between auxin and cold stress responses, we took advantage of the mutants of *Arabidopsis thaliana* with altered response to auxin transport and signal. Screening of the mutants for root growth recovery after cold stress at 4 °C revealed that the auxin signaling mutant, solitary root 1 (*slr1*; mutation in *Aux/IAA14*), shows a hypersensitive response to cold stress. Genome-wide expression analysis of miRs in the wild-type and *slr1* mutant roots using next-generation sequencing revealed 180 known and 71 novel cold-responsive microRNAs. Cold stress also increased the abundance of 26–31 nt small RNA population in *slr1* compared with wild type. Comparative analysis of microRNA expression shows significant differential expression of 13 known and 7 novel miRs in *slr1* at 4 °C compared with wild type. Target gene expression analysis of the members from one potential candidate miR, miR169, revealed the possible involvement of miR169/*NF-YA* module in the Aux/IAA14-mediated cold stress response. Taken together, these results indicate that SLR/IAA14, a transcriptional repressor of auxin signaling, plays a crucial role in integrating miRs in auxin and cold responses.

## 1. Introduction

Cold stress is a serious threat to the sustainability of crop yield. In 2009, the chilling temperature alone resulted in crop damage equivalent to approximately 158 billion yen in Japan. Similarly, early and late frost results in damaging the vegetable and fruit production equivalent to 5–6 billion yen every year in Japan [1]. Cold stress also limits the geographical distribution of many important crop species [2]. In response to cold stress, plants show various phenotypic symptoms, including poor germination, stunted growth, yellowing of leaves (chlorosis), reduced leaf expansion, wilting of leaves, necrosis, and premature death [3]. Cold stress also severely affects the reproductive development of plants [4]. Exposure to stresses leads to changes in complex and interactive cellular and molecular processes required for physical adaptations and increased survival of the plants. Hence, a better understanding of the pathways that regulate plant growth during cold temperature stress is essential.

Hormonal regulation is one of the prime regulatory mechanisms involved in the survival of plants, which is a complex process comprising interactions of various hormones at transcriptional, translational, and cellular levels [5]. Among the hormones, auxin (indole-3-acetic acid, IAA) controls all aspects of plant growth and development, i.e., from embryogenesis to senescence [6]. Consistently, auxin has been shown to play an essential role in regulating plant growth and development under both high- and low-temperature stresses [1,7,8,9,10,11]. In *Arabidopsis*, cold stress seems to affect both auxin transport and signaling processes. Long-term cold stress results in coordinated down- and upregulations of Aux/IAA and ARF protein family, respectively [12], while short-term cold stress affects the polar transport of auxin through modulating GNOM-mediated intracellular cycling of PIN2, a detrimental process for the functionality of PIN proteins [9,13].

It was also demonstrated that cold stress alters the auxin homeostasis and considerably increases the auxin level in the root meristem, resulting in root growth inhibition. Restoring the auxin level through increased transport could restore the root growth even under cold stress [9,13]. Recently, the involvement of auxin maximum in the quiescent center (QC) has been shown to be an essential factor in preserving the root stem cells at a quiescent status under chilling stress [14]. The authors also demonstrated that this chilling stress-specific sacrifice-for-survival mechanism not only protects the stem cell niche from chilling stress but also improves the root’s ability to withstand the accompanying environmental pressures and to recover when ambient temperatures rise to an optimal level [14]. In rice, 4 °C cold stress resulted in 1.2–1.6-fold increase in IAA level [15]. Differential expression of 3 auxin efflux carrier genes, 9 *ARF* genes, and 10 *Aux/IAA* genes was observed under cold stress [16]. The genome-wide analysis of the early auxin-responsive gene families in rice under cold stress revealed both up- and downregulation of several genes of the Gretchen Hagen 3 (*GH3*), *Aux⁄IAA*, small auxin upregulated RNA (*SAUR*), and auxin response factor (*ARF*) families [17]. *OsGH3-2* overexpression-induced increase in cold tolerance was attributed to the combined effects of reduced free IAA content, alleviated oxidative damage, and decreased membrane permeability [18].

Additionally, it has also been shown that several known components of the cold signaling pathway are linked to auxin. For instance, SAP and MIZ1 domain-containing ligase1 (SIZ1), which is a central regulatory component of the cold response pathway and stabilizes inducer of CBF expression 1 (ICE1), is directly linked to auxin-mediated root architecture patterning [19,20]. Another downstream component of cold signaling pathway, nucleoporin 160 (*At*NUP160), which plays a critical role in the nucleocytoplasmic transport of mRNAs under cold stress [21], has also been shown to play an essential role in auxin signaling [22]. Collectively, these results demonstrate the importance of auxin in regulating plants’ cold response. However, what remains obscure are the molecular components that integrate auxin and cold stress response.

MicroRNAs (miRs) are small noncoding RNAs, usually consisting of 20–22 nucleotides for animals and 20–24 nucleotides for plants, that have emerged as ubiquitous post-transcriptional gene regulatory molecules [23]. miRs bind to complementary mRNA molecules and act as negative regulators of gene expression through endonucleolytic cleavage or translational repression of the cognate mRNA targets [24]. Lately, miR regulation of the stress response pathway has been established in several plant species [25,26,27,28]. Consistently, many cold-stress-responsive miRs have been identified in various plant species with variable results in different reports [27,29,30]. Upregulation of miRs, namely miR168, miR169, miR172, miR393, miR397, and miR395 during cold stress has been commonly observed in several plant species [31]. On the other hand, Lv et al. (2010) identified 18 cold-responsive miRs in rice, of which most were downregulated, indicating that the up- and/or downregulations of target genes controlled by miRs play an important role in a plant’s adaptation to cold stress [32]. For instance, overexpression of miR408, which targets cuproproteins belonging to the phytocyanin family and laccase, results in cold tolerance [33,34]. Consistently, miR408 knockout lines show a hypersensitive response to cold stress [34]. Overexpression of miR397, which targets laccases and a casein kinase beta subunit 3, also results in increased freezing tolerance after cold acclimation [35]. Overexpression of miR394a and *LCR* have demonstrated the positive role of this miR–target pair in response to low-temperature stress [36]. In rice, miR319 overexpression lines show an increased survival rate under cold stress [37,38]. In trifoliate orange, overexpression of the precursor of ptr-miR396b results in enhanced cold tolerance [39]. Collectively, these results suggest that miRs are potential regulators of cold stress response pathways across the plant species.

Reports on miRs have also demonstrated their strong potentials in modulating auxin signal transduction, and several genes in auxin signaling have been reported as targets of miRs. For example, miR393 targets four closely related F-box genes, including the auxin receptor *TIR1* [40,41]. miR393 also targets a basic helix–loop–helix transcription factor from *Arabidopsis* that is homologous to GBOF-1 from tulip and annotated as an auxin-inducible gene [42]. Interestingly, Liu et al. (2017) showed that the heterologous expression of rice miR393a results in enhanced cold tolerance in switchgrass (*Panicum virgatum* L.) [43]. Some auxin response factors (*ARFs*) have also been reported as targets of miRs [44,45]. *ARF10*, *ARF16*, and *ARF17* are regulated by miR160 [46,47], while miR167 negatively regulates the expression of *ARF2*, *ARF3*, *ARF4*, *ARF6*, and *ARF8* [48]. Few reports show that miR160 and the target *ARFs* are conserved between dicots and monocots [49,50]. Liu et al. (2012) suggested that miR167 is essential for the appropriate expression of at least four *OsARFs* that contribute to the normal growth and development of rice [51]. Additionally, Lv et al. (2010) reported the involvement of miR167 during cold stress in rice, which had also been shown to be involved in regulating auxin signaling through modulating auxin response factors in several plant species [32,51,52,53,54]. Moreover, miR164 fine-tunes the auxin signals by targeting the NAC domain transcription factors [55]. Taken together, these findings suggest that miRs could be the potential link in integrating the auxin and cold stress responses.

In the present work, we tried to decipher the miRs that may regulate both auxin and cold stress responses by identifying auxin mutant that shows altered response to root growth after cold stress, followed by comparative analyses of genome-wide miRs in the wild type and a cold-stress-sensitive auxin mutant by deep sequencing. Our results revealed that *Aux*/*IAA14* mutant *slr1* shows a hypersensitive response to cold-induced root growth inhibition. Comparative microRNA expression analysis displayed significant differential expression of 13 known and 7 novel miRs in *slr1* during cold stress compared with the wild-type. Interestingly, the majority of the differentially expressed miRs were downregulated in *slr1* in comparison to the wild-type. The alteration of a significant number of miRs at 4 °C in the *slr1* background suggests that SLR/IAA14 plays a crucial role in integrating cold and miR responses. Further, expression analysis of the target genes of one of the potential candidates, miR169, revealed that the miR169/*NF-YA* module may play an important role in integrating IAA14-mediated auxin signaling, miR, and cold stress.

## 2. Results

### 2.1. Auxin Signaling Mutant slr1 Is Susceptible to Low Temperature

**Figure 1:**
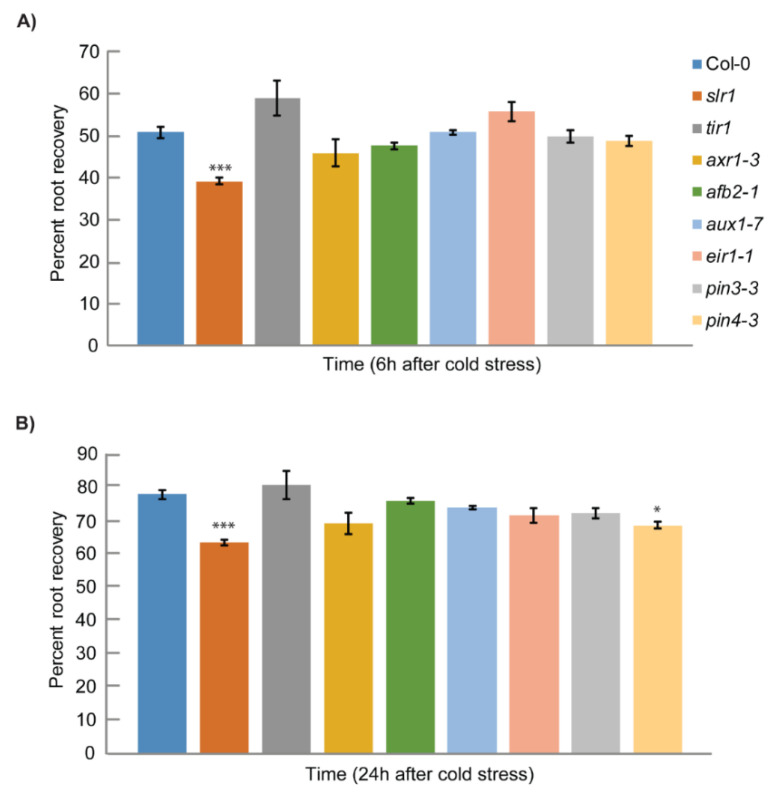
Screening of auxin mutants for root growth recovery response after cold stress: (**A**) percentage root growth recovery at 6 h after 12 h of cold stress; (**B**) percentage root growth recovery at 24 h after 12 h of cold stress. Root growth recovery was calculated against the root growth at 23 °C. Vertical bars represent mean ± S.E. Data are from at least three independent experiments (*n* = 3 or more) with 8–10 seedlings per treatment. Asterisks denote the statistical significance between control and treatment as judged by the Student’s *t*-test (* *p* < 0.05 and *** *p* < 0.001).

**Figure 2:**
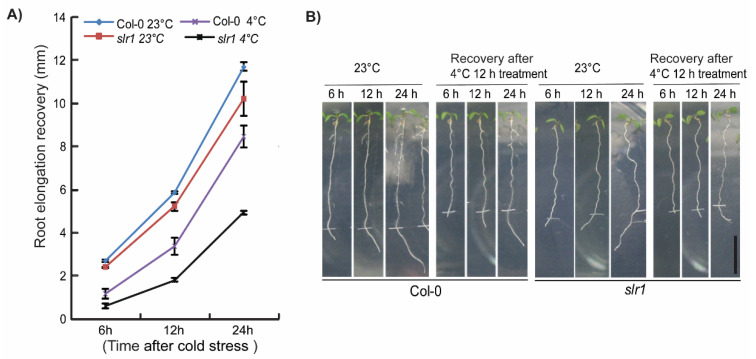
Auxin mutant *slr1* shows hypersensitive response to cold stress: (**A**) Comparison of primary root elongation at optimal temperature (23 °C) and 12 h cold-stressed seedlings in wild type (Col-0) and *slr1*. Note that slr1 shows slower root growth recovery compared with wild type at all time points we tested. Vertical bars represent mean ± SE. Data are from at least three independent experiments (*n* = 3 or more) with 8–10 seedlings per treatment. slr1 root growth recovery at 4 °C was statistically significant at all time points as judged by the Student’s *t*-test. (**B**) Root phenotype of Col-0 and *slr1* during 24 h recovery period after cold stress at 4 °C for 12 h. Tick marks indicate the starting point of the recovery at 23 °C. Scale bar = 10 mm.

To better understand the role of auxin in cold stress response, we first focused on identifying the cold-responsive auxin mutants. The screening of the auxin mutants was performed based on the root growth recovery assay developed in our lab earlier [9]. Auxin signaling mutants *slr1*, *tir1*, *axr1-3*, and *afb2-1* and auxin transport mutants *aux1-7*, *eir1-1*, *pin3-3*, and *pin4-3* were subjected to cold stress screening [56,57,58,59,60,61,62,63]. The root elongation recovery was analyzed after 6 and 24 h of recovery (Figure 1) [9]. Consistent with previous results, we also found that cold stress inhibits root growth recovery by approximately 50% in the wild type after 6 h (Figure 1A). Through root growth recovery screening, we could identify *slr1*, an Aux/IAA14 mutant as a potential candidate as *slr1* showed slower root elongation recovery at both 6 and 24 h time points (Figure 1, Supplementary Figure S1). The other mutants did not show any significant difference compared with wild-type for root elongation recovery except auxin transport mutant *pin4-3*, which showed a slight but statistically significant slower root recovery response at 24 h (Figure 1). To further confirm whether *slr1* response to cold stress persists for a longer time, we measured the root growth recovery till 24 h and found that *slr1* showed slower root growth recovery at all time points tested (Figure 2A,B).

### 2.2. High-Throughput Sequencing of Small RNA Libraries

**Figure 3:**
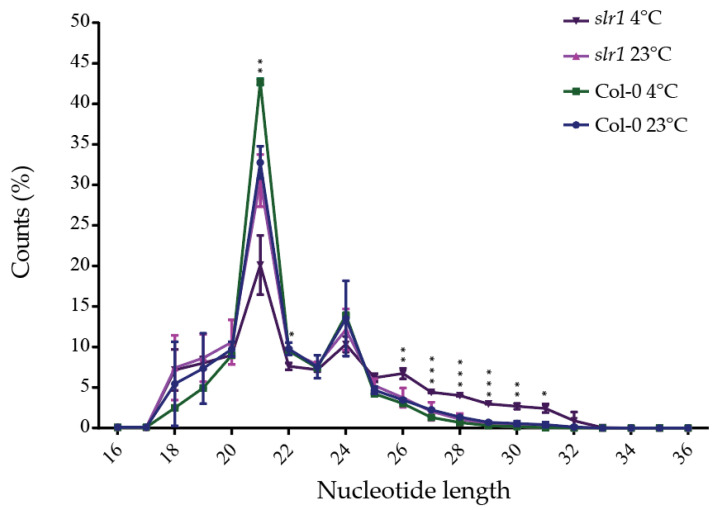
Nucleotide length distribution in wild type (Col-0) and *slr1*. Nucleotide length distribution of small RNA libraries. The results are obtained from two independent biological replicates. Vertical bars represent mean ± SD. Asterisks represent the statistical significance between the treatments as judged by the Student’s *t*-test (* *p* < 0.05, ** *p* < 0.01, and *** *p* < 0.001).

After identifying the auxin response mutant *slr1* that shows hypersensitivity to cold-stress-induced root growth, we next focused on identifying the miRs that are responsive to auxin signaling-mediated cold stress response. For this, we performed a comparative RNAseq analysis between wild type (Col-0) and *slr1* using Illumina high-throughput sequencing platform. After trimming the adaptor and low-quality reads, the sequence reads were generated. A total of 2,556,265 different tags were found from the trimmed reads, comprising 933,996 different tags from Col-0 at 23 °C, 441,338 different tags from Col-0 at 4 °C, 626,812 different tags from *slr1* at 23 °C, and 554,119 different tags from *slr1* at 4 °C (Table 1). The nucleotide length distribution in all the libraries showed that most sequences ranged from 19 to 29 nt in size. We found that 21-nt-long small RNAs were the most abundant in all four libraries, followed by 24 nt. The total abundance of the 21 and 24 nt small RNA population types was 46.26% in Col-0 at 23 °C, 56.44% in Col-0 at 4 °C, 42.66% in *slr1* at 23 °C, and 30.43% in *slr1* at 4 °C (Table 2). The significant change in small RNA abundance in the cold-stressed *slr1* compared with the wild-type suggests the importance of auxin signaling in miR-modulated cold stress response. The populations of 26–31 nt small RNAs were drastically affected in *slr1* background by cold stress as an increase in 26–31 nt was observed (Table 2). We also found a significant decrease in the 21 nt small RNA population in *slr1* under cold stress, while this population was increased in Col-0. These results further reinforce the idea that auxin signaling directly regulates miR expression (Figure 3, Table 2). We did not observe any significant changes in 24 nt reads for treatments and genotypes (Table 2, Figure 3). The significant change in the major population of small RNAs in the *slr1* mutant under cold stress confirms the involvement of auxin response in regulating miR functions linked to the cold stress response pathway, and it also provides a possible explanation for the hypersensitive response of *slr1* to cold stress.

### 2.3. Identification of Known and Novel miRs

**Figure 4:**
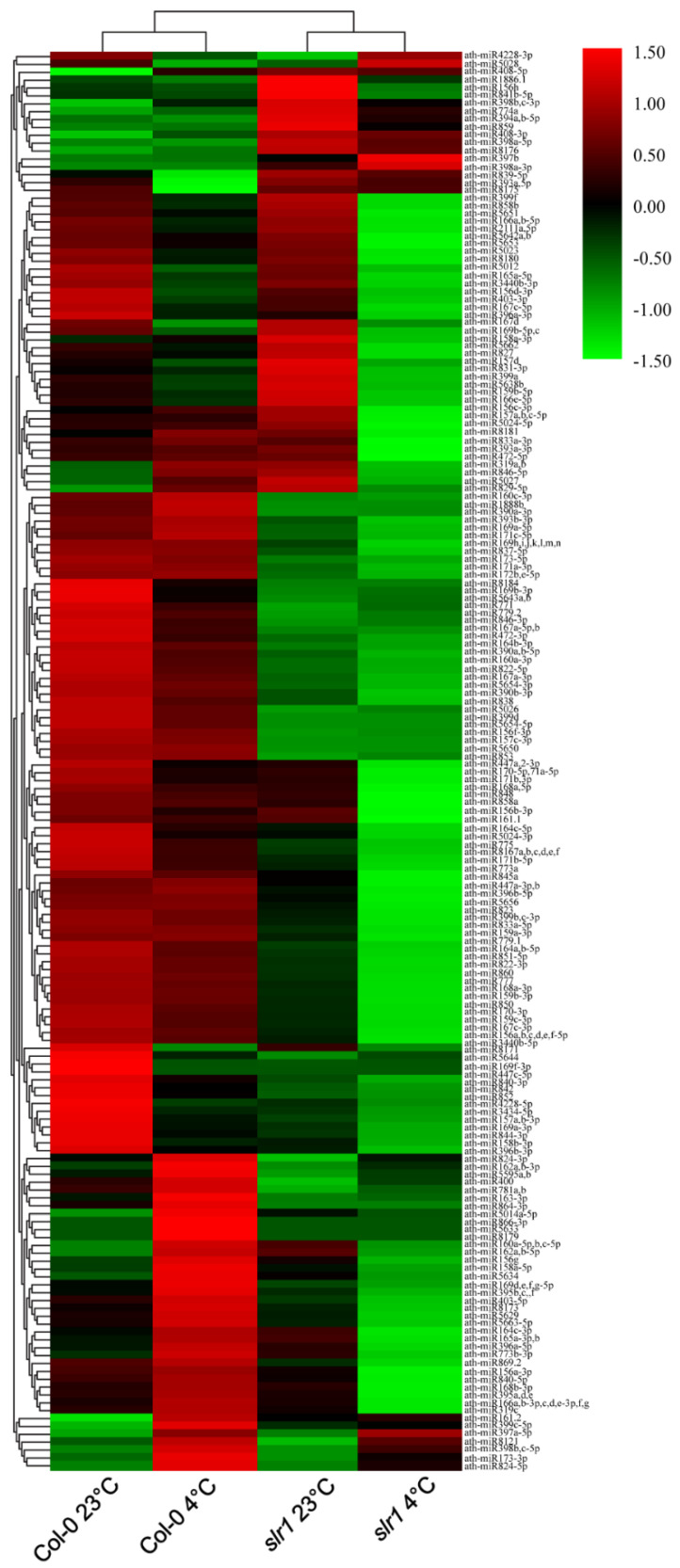
Known miR expression profiles in the root of wild type (Col-0) and *slr1*. The heat map shows the hierarchical cluster analysis of miRs regulated in the cold-stressed root of wild type and *slr1*. The color bars of the heatmap represent the gradient scale of normalized log2-TPM values for each miR. Red color indicates a high level of miR abundance, and green color indicates low abundance. The analysis was performed using two independent biological replicates.

**Figure 5:**
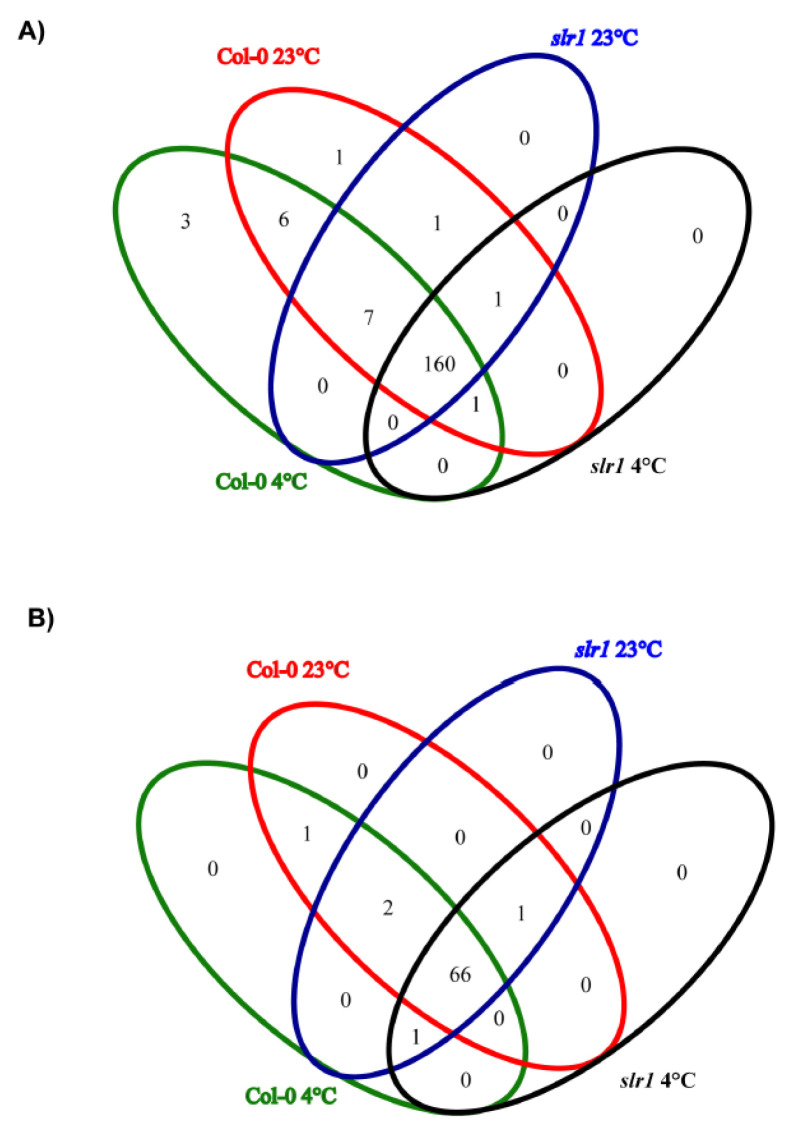
Venn diagram analysis showing the overlap of miRs among four libraries. (**A**) Known miRs identified in auxin-mediated cold stress response. (**B**) Novel miRs identified in auxin-mediated cold stress response.

A total of 180 known miRs, representing 70 families, were identified in eight small RNA libraries made from root tissue (Figure 4 and Figure 5, Supplementary Table S1). In all the eight libraries, the miR166 family was the most abundant, followed by miR165 and miR168 families. During low-temperature stress, we found differential expression patterns of miRs in the wild type and *slr1* (Figure 4, Supplementary Table S1). The comparative analysis between cold-treated wild type and *slr1* revealed that the expression of 13 miRs significantly changed in *slr1* during cold stress (Table 3). We also observed altered expression of 10 miRs in *slr1* background between control and cold treatment (Table 3). Interestingly, the expression of all the miRs, except for one, was downregulated in *slr1* mutant compared to wild type.

For the identification of novel miRs, mirDeep-P pipeline was used. A total of 71 sequences were predicted to be potentially novel miRs from unannotated small RNAs (Supplementary Tables S2 and S3, Supplementary Figure S2). The abundance of novel miRs was lower compared to conserved miRs, and their length varied from 19 to 30 nt. The comparative analysis of novel miRs between Col-0 and *slr1* revealed that seven of them significantly changed in *slr1* under cold stress (Table 4, Supplementary Figure S2).

### 2.4. Validation of miR Expression Patterns

**Figure 6:**
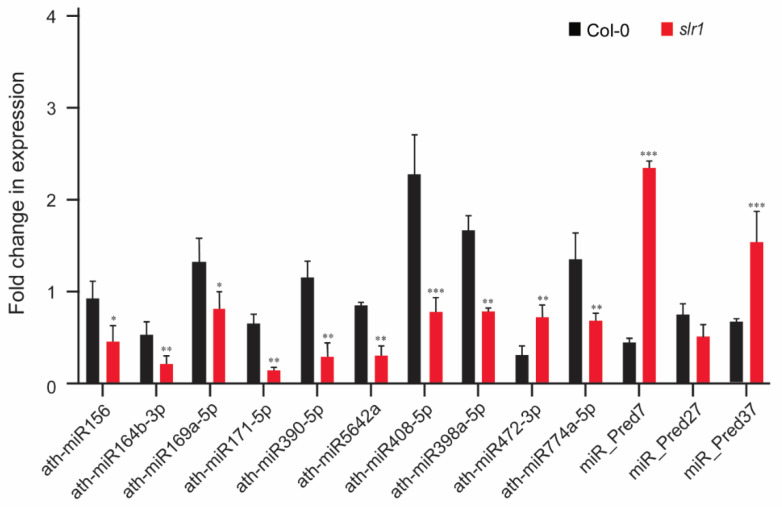
Validation of miR expression in response to cold stress. RT-qPCR validation of selected miRs from cold stress NGS library. Vertical bars represent the mean ± SE of three biological replicates. Asterisks represent the statistical significance between control and treatment as judged by the Student’s *t*-test (* *p* < 0.05, ** *p* < 0.01, and *** *p* < 0.001).

The consistency in miR expression identified by deep sequencing was validated using quantitative real-time PCR. We selected 13 differentially expressed miRs that were regulated in response to cold in the root. The validation was performed using 10 known miRs (miR156, miR164b-3p, miR169a-5p, miR171-5p, miR390-5p, miR5642a, miR408-5p, miR398a-5p, miR472-3p, and miR774a-5p) and 3 novel miRs (miR_Pred7, miR_Pred27, and miR_Pred37). With the exceptions of miR472 and miR_Pred7, qRT-PCR validation results of the expression of all other miRs showed a trend similar to that of the deep sequencing data, confirming that the observed differences of miR expression using three biological replicates are fairly consistent and reproducible (Figure 6).

### 2.5. miR Target Prediction

**Figure 7:**
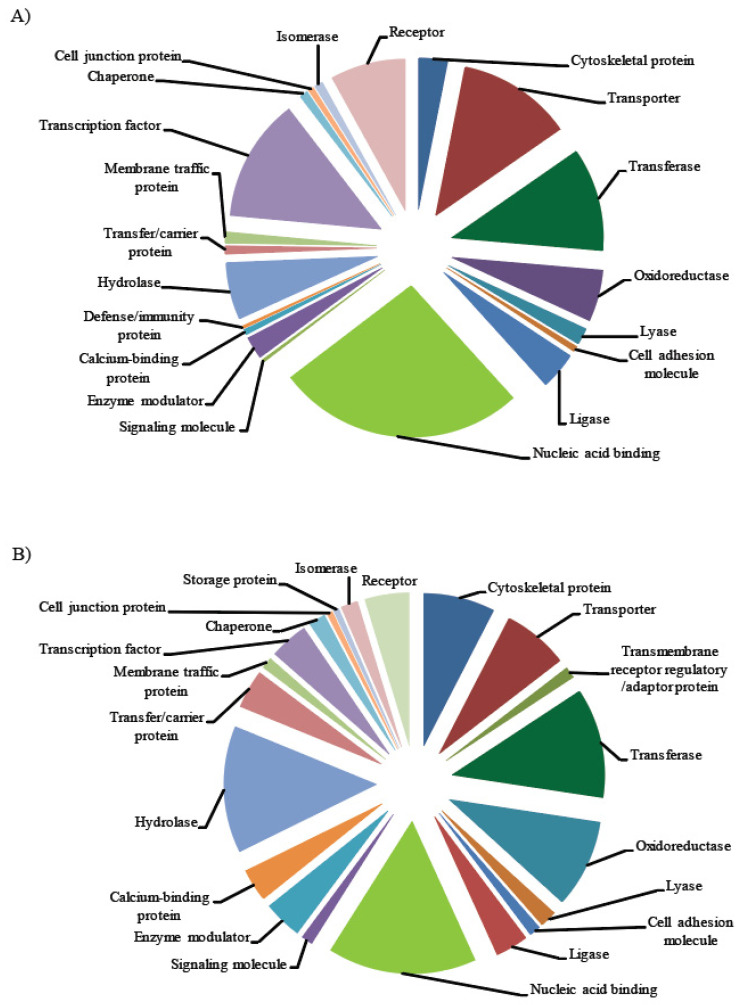
Pie charts showing the classes of proteins targeted by identified miRs: (**A**) protein classes targeted by known miRs; (**B**) protein classes targeted by novel miRs.

The degree of sequence complementarity between miR and its binding site within the target determines the mode of action of miR. High sequence complementarity results in cleavage of targets [47,64,65], while low sequence complementarity results in translational inhibition [66,67]. Several online resources, such as psRNATarget, use the same strategy to identify the plant miR targets. We carried out target prediction to understand the function of identified miRs by using psRNATarget server with preset values. The predicted targets for these miRs were from different classes of proteins associated with development, transport, auxin regulation, signaling, and stress response (Figure 7, Supplementary Tables S1 and S2). For instance, cold stress response and signaling related proteins were targeted by miRs such as miR396b-3p, which targets MYB-like transcription factors, and miR156, which targets *SQUAMOSA promoter-binding protein-like (SPL)* transcription factor (Table 5). miRs like miR390a/b-5p target the TASI-ARF, which is involved in auxin signaling. The possible roles of the target proteins in integrating auxin and cold stress responses are discussed in detail in the discussion section. In general, we predict that the regulation of essential proteins contributing to cold stress tolerance in *slr1* is possibly linked to the cold-susceptible phenotype of the mutant.

### 2.6. miR169/NF-YA Module Is Altered in slr1 under Low-Temperature Stress

**Figure 8:**
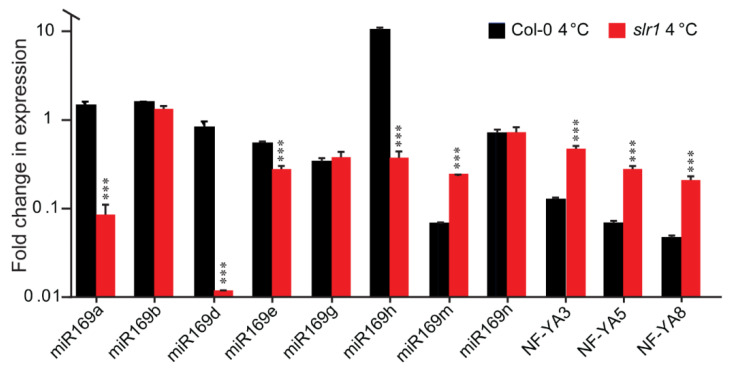
Relative expression of miR169 precursors and *NF-YA3*, *NF-YA5*, and *NF-YA8* in Col-0 and *slr1* after 12 h of cold (4 °C) stress. Vertical bars represent the mean ± SE of three biological replicates. Asterisks denote the statistical significance between the Col-0 and *slr1* at 4 °C as judged by the Student’s *t*-test (*** *p* < 0.001).

To understand the biological significance of the RNAseq results, we selected one of the potential miR candidates, miR169, which is evolutionarily conserved, reported to be present in various plant species including monocots, dicots, ferns, and gymnosperms [68,69,70], and has been shown to be a central regulator of various abiotic stresses, including drought, salt, cold, heat, oxidative, and hypoxic stresses [71]. The miR169 family of *Arabidopsis* has 14 members that mature into four types of different isoforms, differing only by one or two nucleotides [71]. Phylogenetic analysis of miR169 revealed that apart from miR169a, b, c, and h, there are three obvious clades: clade I (mir169d, e, f, g), clade II (miR169i, k, m), and clade III (miR169j, l, n) [71]. The miR169 family members show distinct temporal and differential expression patterns and thus regulate diverse target genes [71,72]. One of the major targets of miR169 for abiotic stress response is nuclear factor Y (NF-Y), a heterotrimeric transcription factor composed of NF-YA, NF-YB, and NF-YC proteins [73]. The link of the NF-Y family members in regulating plant developmental and stress response pathways has been demonstrated in several studies [73,74,75,76,77,78,79,80,81]. Earlier, a direct effect of temperature on miR169h and *NF-YA* was demonstrated [82]. miR169h abundance is directly influenced by temperature; while the abundance was high at high temperature, the abundance was considerably low at low temperature. As expected, miR169 target gene *NF-YA* expression was reciprocal to the abundance of the miR169 expression [82]. They further demonstrated that the NF-Y complex regulates the temperature-dependent flowering and petiole length through directly binding to the promoters of flowering regulator *FT* and the auxin biosynthesis gene *YUC2*. These results make an elegant model linking miR169, NF-Y, and auxin. We tested whether a similar module works for cold stress response in *slr1*. For better clarification of the role of the miR169 family, we selected at least one member from each clade, as well as miR169a, b, and h. Besides miR169m, in cold-stressed *slr1*, all the tested miR169 members showed either a significant reduction in expression (miR169a, miR169d, miR169e, mir169h) or no changes in expression (miR169b, miR169g) (Figure 8). The expression data suggest that among miR169 family members, miR169a, miR169d, and miR169h function as major regulators linking auxin response and cold.

Next, we investigated whether the cold-stress-induced change in miR169 affects the NF-YA abundance reciprocally. In the wild type (Col-0), under cold stress, we observed a decrease in NF-YA abundance (8-, 15-, and 22-fold decreases compared with 23 °C for *NF-YA3*, *NF-YA5*, and *NF-YA8*, respectively), while in *slr1*, there was a higher accumulation of *NF-YA* transcripts compared with the wild type (3.94-, 4.3-, and 4.4-fold increases for *NF-YA3*, *NF-YA5,* and *NF-YA8*, respectively (Figure 8)). Based on the above findings, we speculate that the altered miR169/*NF-YA* module in *slr1* possibly contributes to its cold-stress-susceptible phenotype. Further, the miR169/*NF-YA* module might play a pivotal role during cold stress recovery, and miR169 regulates the expression of this module in an SLR-dependent manner.

## 3. Discussion

Several studies indicate that auxin and microRNAs play essential roles in plant cold stress response [27,30,83,84,85]. Auxin plays a pivotal role in regulating the temperature stress response, and high-temperature stress response directly affects the auxin biosynthesis through altering the expression of phytochrome interacting factors (PIFs) [86]. More recently, phytochromes have been shown to function as thermosensors in *Arabidopsis* [87,88]. Additionally, auxin transport and auxin signaling have also been shown to be altered in response to high temperature [10,89,90]. Under cold stress, polar and lateral auxin transports are altered, resulting in slower growth [1,9,91]. As for microRNAs, a large number of miRs have been implicated in regulating the low-temperature response in different plant species [30,92,93,94,95]. Although these studies represent links (1) between auxin and cold stress and (2) between miR and cold stress, the mechanistic link between microRNA and auxin in regulating plant cold stress response remains elusive. Here we report that (1) the downstream auxin signaling response is crucial for miR-mediated cold stress response in *Arabidopsis* root, (2) the loss of auxin response resulted in altered expression of specific miRs under cold stress, (3) the hypersensitive response of *slr1* to cold stress is possibly linked to the differential expression of cold-regulated miRs, and (4) the miR169/*NF-YA* module possibly plays a major role in integrating auxin signaling, miR, and cold stress.

Intracellular auxin response and miR-mediated gene expression tightly regulate the root growth developmental process in plants. For instance, root elongation is inhibited by the accumulation of auxin in the cell elongation zone [96]. Similarly, the timely regulation of expression of several genes by miR is indispensable for proper root growth [97,98]. It was previously reported that the cold-induced inhibition of root growth is linked to the accumulation of auxin in the root meristematic zone, which results from the GNOM-regulated dysfunction of auxin efflux carrier, PIN2 [9]. In rice, genes linked to auxin signaling such as *Aux/IAA* and *ARF* were found to be altered by cold stress [16,17]. Consistently, in our current screening, we found *slr1* to show a hypersensitive response to cold treatment during the root elongation recovery process. *SLR* encodes IAA14 protein, which functions as a repressor for auxin-induced gene expression, and a downstream auxin signaling component [56]. Mutation in *slr1* results in stable IAA14 protein, which does not degrade in response to IAA, resulting in auxin insensitivity [99]. The finding that *slr1* shows a hypersensitive response to cold stress indicates the importance of the downstream auxin signaling pathway in the process.

miR modulates the plant development, plant response to various environmental challenges, and auxin response by regulating the gene expression post-transcriptionally. From the beginning of plant miR study, its role in low-temperature and other stresses has been reported [25,100,101,102]. Previous studies suggest that during stress conditions, an increase in miR expression deregulates the negative regulators of stress. In contrast, a decrease in miR expression leads to the accumulation of positive regulators of stress [103,104]. The significant reduction in the expression of several miRs in the *slr1* mutant compared to the wild type suggests that there could be an increased activity of negative regulators in the mutant leading to a susceptible phenotype (Table 3). The observed differences in post-transcriptional regulation of *NF-YA* transcripts by miR169 in wild type and *slr1* under cold stress supports this notion (Figure 8). The miR169/*NF-YA* module, which is one of the stress-regulated miR target modules, showed an altered expression pattern in *slr1* under cold stress. Our results demonstrate that miR169a, miR169d, and miR169h are the primary targets of cold in the *slr1* mutant (Figure 8). This is consistent with the idea that the temporal and differential expressions of the members within the same microRNA family widely varies depending on the growth stages and acquired stresses [105]. miR169m showed a completely opposite expression pattern in *slr1* under cold stress compared to other members. This is interesting, but the significance of this result is unknown at present. The reduced expression of miR169a, miR169d, and miR169h directly influenced the expression of *NF-YA* transcripts, resulting in higher accumulation in *slr1* than in the wild type under cold stress (Figure 8). The miR169/*NF-YA* module has already been shown to be linked to the regulation of the temperature response in previous studies [82,106,107]. In addition, NF-YA has been shown to modulate auxin response by regulating its biosynthesis [82]. Taken together, these findings suggest that the miR169/*NF-YA* module, which functions in an SLR/IAA14-dependent manner, could function as a major regulator integrating auxin, miR, and cold stress response.

Besides miR169, several other miRs may be involved in regulating the auxin-mediated cold stress response. Low-temperature stress also significantly decreased the expression of miR390 in *slr1* mutant compared to the wild type. miR390 has been suggested to direct the production of tasiRNAs from *trans-acting siRNA3 (TAS3)* transcripts, which regulate the *ARF* genes essential for auxin signaling [108]. Recently, it has been demonstrated that the miR390*/TAS3/ARFs* module plays a key role in regulating lateral root development in salt-stressed poplar through modulating the auxin pathway [109]. Moreover, miR390b-3p targets *AtVps11*, an essential component for endosome organization, intracellular protein transport, vacuole biogenesis, and pollen tube growth [110]. It also targets clathrin heavy chains, which mediate endocytosis and intracellular transport and are required for proper polar distribution of PINs [111]. Differentially regulated miR390 in wild type and *slr1* under low-temperature stress may also link auxin signaling and miRs during the low-temperature stress response.

The essential amino acid tryptophan (Trp) is well known for its requirement in auxin biosynthesis. The plant needs Trp for the synthesis of various proteins and many metabolites. Interference in Trp biosynthesis leads to various developmental defects in plants [112]. The targeting of tryptophan synthetase by miR5642 under low temperature suggests possible crosstalk of low temperature and auxin biosynthesis during the cold stress response. Moreover, the miR774a, which targets several F-box proteins, could also attenuate the auxin signaling as several F-box proteins play indispensable roles in auxin signaling and response [113]. Taken together, these results confirm a complex triangular relation of miR, cold, and auxin response.

Intriguingly, the differentially regulated miRs target a wide range of proteins involved in response to the stimulus, developmental processes, cellular component organization and biogenesis, biological regulation, cellular processes, metabolic processes, and stress signaling (Supplementary Table S1, Figure 7). Although many of the proteins targeted by specific miRs are still functionally not characterized, few of the target proteins have already been reported for their role in regulating various processes, including abiotic stresses such as cold [30,104]. Characterization of the other target proteins in this list will reveal the functional significance of these altered miRs in integrating auxin response and cold stress.

In agreement with the notion that the miRs are often expressed at a lower level, the majority of the novel miRs displayed a lower expression compared to known miRs (Supplementary Table S2). As per miRBase 22.1, these miRs have not been described previously in *Arabidopsis*, which could be due to their low, nondetectable expression level. In the present study, we possibly discovered most of the miRs in *Arabidopsis* during cold stress. The novel predicted miRs and known miRs, namely miR5656, miR774a, and miR8181, that show altered expression patterns in wild type and *slr1* target the transposable elements (TEs) and transposons (Table 5 and Supplementary Table S2). TEs can have a myriad of effects when they are inserted into new locations [114,115,116]. These effects vary depending on the sequence of the TE and the precise location of its insertion. TEs are also responsive and susceptible to environmental changes. Stress-activated TEs might generate the raw diversity that species require over evolutionary time to survive stressful situations [117]. From bacteria to mammals, TE-induced mutations are associated with environmental adaptations (for a review, see the work of E. Casacuberta and J. Gonzalez [117]). In plants, TE-induced mutations result in adaptation to high altitude in soybean, adaptation to changing light environment in *Arabidopsis*, and adaptation to a wide range of environments in wheat [118,119,120,121]. Interestingly, TEs have also been shown to contribute to duplication of *Aux/IAA* genes in soybean [122]. The findings that the cold stress stimulates miRs that potentially target TEs in *slr1* suggest a possible involvement of TEs in integrating auxin and cold stress responses and need further studies.

## 4. Materials and Methods

### 4.1. Plant Materials and Growth Condition

All lines are in the Columbia background of *Arabidopsis thaliana* (L.) Heynh., and Col-0 plants were used as wild type in the present study. *axr1-3*, *aux1-7*, *eir1-1*; *pin3-3*, *pin4-3*, and *tir1-1* were obtained from the Arabidopsis Biological Resource Center (Columbus, OH, USA). *afb2-1* was a kind gift of Nihal Dharmasiri (Texas State University, San Marcos, TX, USA). *slr1* was described in Fukaki et al. (2002) [56].

Surface-sterilized seeds were placed in round, 9-cm Petri plates on modified Hoagland medium [1,9,123] containing 1% (*w*/*v*) sucrose and 1% (*w*/*v*) agar. The plates were kept at 4 °C in the dark for 2 days for seed stratification. After stratification, the plates were transferred to the growth chamber (LPH-220S, NK System, Japan) at 23 °C under continuous white fluorescent light at an intensity of 100 μmol m^−2^ s^−1^, and seedlings were grown vertically for 5 days.

### 4.2. Cold Stress Treatment and Analysis of Root Growth Recovery

Cold stress treatment and growth recovery were performed as described earlier [9]. A 12 h cold treatment at 4 °C typically results in approximately 40–50% root elongation inhibition during the recovery phase at 23 °C [9]. Hence, we selected 12 h incubation at 4 °C as an optimal treatment for analyzing the effect of cold stress. Briefly, 5-day-old seedlings were transferred to new plates containing the modified Hoagland medium and kept at 4 °C for 12 h in the growth chamber (NK System; LH-1-120.S). After cold stress, plates were put back at 23 °C for recovery, whereas the control plates were kept continuously at 23 °C. Percentage root elongation recovery compared to control (grown at 23 °C) of each genotype was used as a measure of screening. The experiments were repeated at least three times, with eight seedlings per treatment. To measure cold stress recovery, seedlings were photographed by a digital camera (Canon Power Shot A640, Canon, Tokyo, Japan), and root growth recovery was analyzed by ImageJ1.47t software (http://rsbweb.nih.gov/ij/).

### 4.3. Chemicals

Difco Bacto Agar was purchased from BD Biosciences, San Jose, CA, USA. Other chemicals were from Wako Pure Chemical Industries, Osaka, Japan.

### 4.4. Small RNA Isolation and Sequencing

After 5-day-old seedlings grown on modified Hoagland media were cold-treated at 4 °C for 12 h or grown at 23 °C as control, their root tissues were used to construct small RNA libraries. Briefly, the total RNA was isolated from the roots of cold-stress-treated and control samples by using the RNeasy Kit (Qiagen, MD, USA) according to the manufacturer’s instructions, and small RNA was enriched by RNeasy MinElute Cleanup kit (Qiagen, MD, USA). Eppendorf BioPhotometer plus (Hamburg, Germany) was used to detect the quality and the concentration of RNA. Construction of the sRNA libraries and deep sequencing were carried out by BGI (Beijing, China). Briefly, RNA with lengths of 16–36 nt was separated and purified using denaturing polyacrylamide gel electrophoresis, followed by sequential 3′ and 5′ RNA adaptor ligation to the small RNAs using T4 RNA Ligase. The adaptor-ligated samples were then reverse transcribed and amplified by PCR to construct the final libraries. Eight small RNA libraries—four from the wild type (two each from 23 °C and 4 °C) and four from auxin mutant *slr1* (two each from 23 °C and 4 °C)—were prepared and subjected to high-throughput sequencing on Illumina platform (Illumina HiSeq 4000 platform, Illumina, San Diego, CA, USA). The sequencing resulted a total of 270,303,139 reads (47,318,921 and 39,919,856 reads from Col-0 at 23 °C; 30,230,648 and 23,124,483 reads from Col-0 at 4 °C; 22,247,497 and 43,063,180 reads from *slr1* at 23 °C; 25,807,408 and 45,591,146 reads from *slr1* at 4 °C).

### 4.5. Data Deposition Information

The sequencing data that support the findings of this study have been deposited in the NCBI SRA database with the SRA accession code PRJNA579274. The SRA record is accessible through the following link: https://www.ncbi.nlm.nih.gov/sra/PRJNA579274.

### 4.6. Bioinformatic Analysis of the sRNA Sequencing Data

After sequencing, the raw reads were filtered, and adapter sequences were removed along with contamination and low-quality reads from raw reads. The remaining unique sequences (clean reads) were then processed to identify known and novel microRNAs.

Known miRs from *Arabidopsis* root after cold stress were identified using CLC Genomics Workbench v12.0 (CLC Bio, Aarhus, Denmark). Briefly, clean reads processed from raw sequencing reads after trimming adaptor sequences and removing low-quality reads were further analyzed by CLC workbench to extract and group sRNA. Sequences shorter than 16 nt and larger than 36 nt along with noncoding RNA such as rRNA, tRNA, and snRNA were excluded from further analysis. The remaining reads were and then annotated to identify the known *Arabidopsis* miRs. To identify known miRs, small RNA sequences were annotated against miRBase 22.1 (http://www.mirbase.org/index.shtml) using CLC Genomics Workbench 12.0 based on their sequence homology. Finally, the mapped miRs were obtained, which then were normalized using the reads per million reads (RPM) method. Normalized reads were then used to determine the fold change between the control and stressed samples.

Novel miRs were identified by using mirDeep-P, a plant-specific miR identification pipeline [124]. Briefly, for each sequenced small RNA library, reads were filtered by length, and only those between 16 and 36 nt were retained. FASTA-formatted reads were then analyzed by miRDeep-P using the *Arabidopsis* genome as a reference.

### 4.7. Prediction of miR Targets

The targets of identified miRs were predicted using psRNATarget (the plant small RNA target server, 2011 release; http://plantgrn.noble.org/psRNATarget/) by aligning with *Arabidopsis* transcripts and default parameters which included a threshold cut-off of 3.0, a complementarity scoring length of 20 bp, and the energy required for target accessibility equal to 25 kcal/mole.

### 4.8. Quantitative RT-PCR Validation of Selected Differentially Expressed miRs

Analysis of miR expression was performed using the poly(T) adaptor RT-PCR method by Mir-X miR First-Strand Synthesis kit (Clonetech, Takara Bio, Moutain View, CA, USA) as per manufacturer’s instruction. Briefly, for polyadenylation and cDNA synthesis, 1 μg of DNaseI-treated total RNA was incubated at 37 °C for 60 min in a 10 μL reaction volume containing mRQ enzyme, and then the reaction was terminated at 85 °C for 5 min to inactivate the enzymes. Quantitative RT-PCR (qRT-PCR) was run on a TaKaRa Dice Real-Time apparatus (Takara, Shiga, Japan) with the SYBR Green I Master kit (Bio-Rad, Hercules, CA, USA). The reaction conditions for qRT-PCR included the following steps: 10 s at 95 °C followed by 40 cycles of denaturation for 10 s at 95 °C and annealing for 20 s at 60 °C, and extension for 15 s at 72 °C. miRs were quantified using specific primer pairs with the translation initiation factor elongation factor 1-α (*EF1α*) as the normalization control. Relative transcript abundance was calculated using the 2^−ΔΔCT^ method [125]. All experiments were performed using three biological replicates and three technical replicates. The miR169 family primers were adapted from Serivichyaswat et al. [126]. All the primers used in the study are listed in Supplementary Table S4.

### 4.9. Statistical Analysis

Results are expressed as the means ± SE from the appropriate number of experiments. A two-tailed Student’s *t*-test was used to analyze statistical significance.

## 5. Conclusions

The present study provides a basic platform to explore the genetic and cellular mechanisms by which auxin and miRs regulate the cold stress response. Identification of a handful of target miRs, including miR169, from the comparative RNA sequencing analyses between the wild type and the auxin signaling mutant *slr1* indicates that auxin-regulated miRs play essential roles in maintaining the cellular signaling system to ensure an optimal cold stress response. Future overexpression or loss-of-function studies with the specific miRs that are altered in *slr1* mutant and their targets will further clarify how the auxin- and miR-mediated pathways contribute to regulating the cold stress response.

## Figures and Tables

**Table 1:** Summary of small RNA libraries after deep sequencing.

Sl. No	Sample Name	Number of Reads	Average Length	Number of Reads after Trim	Percentage Trim	Average Length after Trim
1	Col-0 23 °C_Rep1	47,318,921	21.6	46,240,335	97.72%	21.6
2	Col-0 23 °C_Rep2	38,919,856	22.4	38,635,669	99.27	22.4
3	Col-0 4 °C_Rep1	30,230,648	23.8	30,004,070	99.25%	23.8
4	Col-0 4 °C_Rep2	23,124,483	21.9	22,863,639	98.87%	21.9
5	*slr1* 23 °C_Rep1	22,247,497	21.4	21,471,561	95.51%	21.4
6	*slr1* 23 °C_Rep2	43,063,180	22.1	42,081,401	97.72%	22.1
7	*slr1* 4 °C_Rep1	25,807,408	22.9	24,711,139	95.75	22.4
8	*slr1* 4 °C_Rep2	43,591,146	23.0	43,069,325	98.8%	23.0

**Table 2:** Distribution of frequency percentage of nucleotide length.

	Sample Name	Frequency Percentage of nt Length							
		21 nt	24 nt	26 nt	27 nt	28 nt	29 nt	30 nt	31 nt
1	Col-0 23 °C	32.76	13.50	3.46	2.25	1.37	0.71	0.56	0.41
2	Col-0 4 °C	42.49	13.95	3.02	1.31	0.69	0.30	0.16	0.06
3	*slr1* 23 °C	30.52	12.14	3.75	2.06	1.08	0.48	0.26	0.16
4	*slr1* 4 °C	20.12	10.31	6.72	4.42	4.00	2.97	2.68	2.43

**Table 3:** List of known microRNAs (miRs) whose expression changed significantly during cold stress. Blue color represents the downregulation and red color represents upregulation in the expression in the mutant. Light green color represents Col-0 and light pink color represents *slr1* genotypes respectively. The values in the temperature columns (23 °C and 4 °C) represent the normalized expression values. Data were obtained from two independent biological replicates. Statistical significance between the treatments and genotype was obtained using Student’s *t*-test. ^a^ Denotes miRs showing significantly altered expression between wild type and *slr1* at 4 °C.

miR ID	Col-0			*slr1*			*p*-Value Col-0 vs. *slr1*	
	23 °C	4 °C	*p*-Value	23 °C	4 °C	*p*-Value		
			23 °C vs. 4 °C			23 °C vs. 4 °C	23 °C	4 °C
miR156a,b,c,d,e,f,	114.53	100.9	0.809	78.61	54.51	0.654	0.648	0.002 ^a^
miR164b-3p	22.5	13.47	0.611	3.64	2.79	0.447	0.338	0.006 ^a^
miR169a-5p	26.49	30.81	0.613	15.9	12.7	0.587	0.296	0.059 ^a^
miR170,miR171a-5p	4.7	2.96	0.638	3.17	0.92	0.051	0.652	0.27
miR390a,b-5p	1385	812.14	0.423	383.72	263.68	0.341	0.223	0.029 ^a^
miR390b-3p	65.18	49.73	0.583	25.47	16.48	0.254	0.237	0.029 ^a^
miR396b-3p	6.34	2.97	0.389	2.72	1.38	0.009	0.36	0.018 ^a^
miR398a-5p	0.56	0.48	0.854	2.87	1.88	0.603	0.297	0.013 ^a^
miR399a	4	3.15	0.165	5.65	2.31	0.049	0.148	0.224
miR399b,c-3p	25.8	23.93	0.845	17.41	10.81	0.027	0.413	0.029 ^a^
miR408-5p	14.55	33.15	0.249	42.01	37.83	0.684	0.034	0.764
miR447a-3p,miR447b	21.39	23	0.864	15.29	6.72	0.036	0.43	0.105
miR472-3p	379.33	154.02	0.539	63.04	45.1	0.241	0.41	0.04 ^a^
miR5642a,b	77.64	62.74	0.059	82.11	34	0.239	0.891	0.028 ^a^
miR5656	1.31	1.33	0.982	0.81	0.18	0.044	0.235	0.257
miR773a	419.47	248.54	0.605	167.81	77.04	0.032	0.465	0.019 ^a^
miR774a	0.21	0.3	0.496	0.85	0.51	0.327	0.028	0.495
miR775	26.11	15.41	0.621	9.78	5.68	0.266	0.472	0.04 ^a^
miR8180	0.85	0.5	0.257	0.83	0.13	0.048	0.914	0.257
miR8181	1.49	2.08	0.567	1.98	0.73	0.018	0.25	0.243
miR824-3p	177.08	365.65	0.267	99.59	171	0.037	0.484	0.149
miR850	8.16	6.72	0.685	3.71	1.52	0.103	0.017	0.238
miR852	3.7	1.72	0.468	1.15	0.9	0.384	0.372	0.058 ^a^

**Table 4:** List of predicted miRs whose expression changed significantly during cold stress. Blue color represents the downregulation and red color represents upregulation in the expression in the mutant. Light green color represents Col-0 and light pink color represents *slr1* genotypes respectively. Data were obtained from two independent biological replicates. The values in the temperature columns (23 °C and 4 °C) represent the normalized expression values. Statistical significance between the treatments and genotype was obtained using Student’s *t*-test. ^a^ Denotes miRs showing significantly altered expression between wild type and *slr1* at 4 °C.

miR ID	Col-0			*slr1*			*p*-Value Col-0 vs. *slr1*	
	23 °C	4 °C	*p*-Value	23 °C	4 °C	*p*-Value		
			23 °C vs. 4 °C			23 °C vs. 4 °C	23 °C	4 °C
Pred_7	30.68	16.18	0.521	36.20	69.86	0.033	0.84	0.015 ^a^
Pred_9	27.75	21.95	0.483	17.38	12.98	0.274	0.294	0.007 ^a^
Pred_11	10.06	6.75	0.637	15.59	21.14	0.289	0.506	0.018 ^a^
Pred_19	10.24	6.64	0.271	6.71	4.67	0.434	0.383	0.0031 ^a^
Pred_25	3.38	1.59	0.185	2.15	0.98	0.002	0.305	0.011 ^a^
Pred_26	6.32	5.14	0.647	2.90	1.74	0.503	0.304	0.056 ^a^
Pred_27	4.01	4.49	0.774	3.03	2.17	0.302	0.040	0.280
Pred_35	2.80	1.26	0.099	1.49	0.48	0.071	0.029	0.294
Pred_36	2.19	2.92	0.755	3.08	1.81	0.039	0.107	0.638
Pred_37	2.30	1.41	0.007	3.57	2.38	0.247	0.010	0.313
Pred_44	1.47	0.78	0.015	1.01	0.43	0.466	0.551	0.076
Pred_47	0.99	0.66	0.387	0.30	0.33	0.827	0.012	0.411
Pred_53	0.64	1.03	0.743	1.20	1.27	0.941	0.058	0.868
Pred_60	0.31	0.33	0.835	0	0	0	0.000	0.040 ^a^
Pred_62	0.22	0.14	0.060	0.001	0.147	0.116	0.001	0.983
Pred_65	0.26	0.28	0.966	1.21	0.647	0.475	0.015	0.658

**Table 5:** Predicted miR target list.

miR ID	Target Locus
miR156a,b,c,d,e,f,	SQUAMOSA promoter-binding protein-like 15 (AT3G57920), SQUAMOSA promoter-like 11 (AT1G27360), SPL13B (AT5G50670), SPL9 (AT2G42200), SPL2 (AT5G43270), SPL10 (AT1G27370), SPL13A (AT5G50570), SPL10 (AT1G27370), SBP domain transcription factor (AT1G69170), SPL3 (AT2G33810), protein kinase superfamily protein (AT3G28690), SPL4 (AT1G53160), SPL5 (AT3G15270), transposable element gene (AT1G16660), cysteine/histidine-rich C1 domain family protein (AT2G21840)
miR164b-3p	PPR repeat protein (AT5G14770)
miR169a-5p	Nuclear factor Y, subunit A8 (AT1G17590, AT1G54160)
miR170, miR171a-5p	Transposable element gene (AT2G06790, AT3G30393, AT1G36470, AT1G50860, AT3G29732, AT2G12305), MATE efflux family protein (AT1G15180), F-box family protein (AT5G3946)
miR390a,b-5p	TASIR-ARF (AT5G57735), transmembrane kinase-like 1 (AT3G24660)
miR390b-3p	Vacuolar protein sorting 11 (AT2G05170), RNA-binding (RRM/RBD/RNP motifs) family protein (AT3G07810), transposable element gene (AT2G41570, AT1G35990), clathrin heavy chain (AT3G11130)
miR396b-3p	MYB76 (AT5G07700), ATBTAF1 (AT3G54280), RNA helicase family protein (AT1G58060)
miR398a-5p	SETH2, UDP-glycosyltransferase superfamily protein (AT3G45100); alpha/beta-hydrolases superfamily protein (AT3G48080); ARM repeat superfamily protein (AT5G06120)
miR399a	PHO2/UBC24 (AT2G33770), CYP705A30 (AT3G20940), terpenoid cyclases family protein (AT1G78500), sodium bile acid symporter family (AT2G26900), transposable element gene (AT3G43867), nucleic acid-binding (AT1G27750)
miR399b,c-3p	PHO2 (AT2G33770), wall-associated kinase 2 (AT1G21270)
miR408-5p	Oxidoreductase, 2OG-Fe(II) oxygenase (AT4G02940, AT4G25310), nucleotide/sugar transporter family (AT4G03950), glutathione S-transferase TAU 25 (AT1G17180), leucine-rich repeat protein kinase family protein (AT1G04210), unknown protein (AT4G37030), pseudogene (AT2G31860), calmodulin-binding protein-related (AT5G10660), early-responsive to dehydration stress protein (AT4G35870)
miR447a-3p, miR447b	P-loop containing nucleoside triphosphate (AT5G60760), FAD-dependent oxidoreductase family protein (AT2G22650), lncRNA (AT5G05905)
miR472-3p	Disease resistance protein (CC-NBS-LRR class) family (AT5G43740, AT1G12290, AT1G15890); RPS5, disease resistance protein (CC-NBS-LRR class) family (AT1G12220)
miR5642a,b	Tryptophan synthase beta-subunit 1 (AT5G54810); VHA-E3, vacuolar H+-ATPase subunit E isoform 3 (AT1G64200)
miR5656	Mitochondrial editing factor 9 (AT1G62260), zinc finger (C3HC4-type RING finger) family protein (AT5G60710), transposable element gene (AT3G29787)
miR773a	Remorin family protein (AT3G57540), root FNR 1 (AT4G05390), Acyl-CoA N-acyltransferase with RING/FYVE/PHD-type zinc finger protein (AT4G14920), transposable element gene (transposable element gene), RAD3-like DNA-binding helicase protein (AT1G79950), oleosin3 (AT5G51210)
miR774a	F-box and associated interaction domains-containing protein (AT3G17490), F-box family protein (AT3G19890), transposable element gene (AT1G34405, AT2G01024, AT4G16910, AT3G42996, AT2G07660)
miR775	Dicer-like 1 (AT1G01040), galactosyltransferase family protein (AT1G53290), galactosyltransferase family protein (AT1G53290)
miR8180	Alpha/beta-hydrolases superfamily protein (AT3G55190), plastid division2 (AT2G16070), ATP-dependent helicase family protein (AT2G28240), lncRNA (AT3G08825), fatty acid reductase 1 (AT5G22500),
miR8181	Ovate family protein (AT2G36026), cysteine/histidine-rich C1 domain family protein (AT1G55430), LOB domain-containing protein 39 (AT4G37540), transposable element gene (AT3G30713)
miR824-3p	Concanavalin A-like lectin protein kinase family protein (AT3G08870), pentatricopeptide repeat (PPR)-containing protein (AT5G27300)
miR850	Chloroplast RNA binding (AT1G09340), threonyl-tRNA synthetase (AT2G04842), lncRNA (AT2G08250), AtSWEET4 (AT3G28007)
miR852	IAA-leucine resistant (ILR)-like 1(AT5G56650); IAA-leucine resistant (ILR)-like 2 (AT5G56660); TIR1, F-box/RNI-like superfamily protein (AT3G62980); K+ transporter 1 (AT2G26650); H(+)-ATPase 11 (AT5G62670)

## Supplementary Materials

Supplementary Materials can be found at https://www.mdpi.com/1422-0067/21/22/8441/s1. Figure S1: Root phenotype of seedlings during 24 h recovery period after cold stress at 4 °C for 12 h. Tick marks indicate the starting point of the recovery at 23 °C. Scale bar = 10 mm. Figure S2: Novel miR expression profiles in the root of Col-0 and *slr1*. The heat map shows the hierarchical cluster analysis of novel miRs regulated in the cold-stressed root of Col-0 and *slr1*. The color bars of the heatmap represent the gradient scale of normalized log2-TPM values for each miR. Red color indicates high levels of miRs abundance, and green color indicates low abundance. The analysis was performed using two independent biological replicates. Table S1: Expression of known miRs in small RNA libraries. Table S2: Expression of predicted miRs in small RNA libraries. Table S3: Details of novel miRs predicted in the present study. Table S4: List of primers used in the present study.

## Abbreviations

miRs	MicroRNAs
qRT-PCR	Quantitative Real-Time PCR
EF1α	Elongation Factor 1-α
TEs	Transposable Elements
nt	Nucleotide
IAA	Indole-3-aCetic Acid
ARF	Auxin Response Factor
QC	Quiescent Center
SIZ1	SAP and MIZ1 domain-containing ligase1
PIN	Pin-formed
SAUR	Small Auxin Upregulated RNA
GH	Gretchen Hagen
