# Supplementary material for: Aux/IAA14 Regulates microRNA-Mediated Cold Stress Response in Arabidopsis Roots
Source: Int J Mol Sci. 2020 Nov 10;21(22):8441. doi: 10.3390/ijms21228441 (PMC7697755; doi:10.3390/ijms21228441)
Supplement: Supplementary file 1 [file ijms-21-08441-s001.zip › TableS4.docx]

Supplementary Table S4: List of primers used in present study.

| Sl.No. | Name | Primer Seq 5-->3 |
| --- | --- | --- |
| 1 | ath-miR156 | TGACAGAAGAGAGTGAGCAC |
| 2 | ath-miR164b-3p | CATGTGCCCATCTTCACCATC |
| 3 | ath-miR169a-5p | CAGCCAAGGATGACTTGCCGA |
| 4 | ath-miR171 | TATTGGCCTGGTTCACTCAGA |
| 5 | ath-miR390-5p | AAGCTCAGGAGGGATAGCGCC |
| 6 | ath-miR398a-5p | AAGGAGTGGCATGTGAACACA |
| 7 | ath-miR408-5p | ACAGGGAACAAGCAGAGCATG |
| 8 | ath-miR5642a | TCTCGCGCTTGTACGGCTTT |
| 9 | ath-miR472-3p | TTTTTCCTACTCCGCCCATACC |
| 10 | ath-miR774a | TTGGTTACCCATATGGCCATC |
| 11 | miR_Pred7 | AAGTAAGACTCCGTGGCCCAATGGATAA |
| 12 | miR_Pred27 | TTTGCGGTGCGGAAGAAGTGCGGTGCGGTT |
| 13 | miR_Pred37 | AGGATACTCGGCTCTCACCCG |
| 14 | EF1α-F | CTTGCTTTCACCCTTGGTGT |
| 15 | EF1α-R | TCCCTCGAATCCAGAGATTG |
| 16 | NF-YA3 F | TCTGAGACAGTGGATCTTGGTGCT |
| 17 | NF-YA3 R | ACACTGAAGTTACAACAAGCGAAAG |
| 18 | NF-YA5 F | GGCACATGAGAAGACCTTGCTCCG |
| 19 | NF-YA5 R | ACCGATAACATAAGTAGCCAAGGA |
| 20 | NF-YA8 F | AGGGAAGTCATCCTTGGCTGCT |
| 21 | NF-YA8 R | GTTGAAGCTTGAAGGTTTTTGGTCA |
| 24 | MIR169a pre F | AAAGTAACATGATCGGCAAGTTGTCC |
| 25 | MIR169a pre R | GCGACACAAAGTAACGTGTAGCC |
| 26 | MIR169b pre F | CCCAACGGAGTAGAATTGCATGAAGTG |
| 27 | MIR169b pre R | GCCGAAGGACAACTTGCCAGAAATATG |
| 28 | MIR169d pre F | CAAGGATGACTTGCCGATGTTATCAAC |
| 29 | MIR169d pre R | AAAGAAGGAAACAGAGCCAAGGTCAAC |
| 30 | MIR169e pre F | AGGATGACTTGCCGATTTTCTCAACG |
| 31 | MIR169e pre R | AAAGAAGGGAAGGAAACAGAGCCAAAG |
| 32 | MIR169g pre F | GCATGGAAGAATAGAGAATGAGG |
| 33 | MIR169g pre R | GCCGGACACCAGAATCAGTT |
| 34 | MIR169h pre F | TGTGTGGTAGCCAAGGATGACT |
| 35 | MIR169h pre R | AGCCAAGGAGACTGCCTGACGA |
| 36 | MIR169m pre F | GTAGCCAAGGATGACTTGCCTGTTTC |
| 37 | MIR169m pre R | GGATAGCCAAGGAGACTGCCTGATG |
| 38 | MIR169n pre F | AGAGAGGTCTAACATGGCGGAAAGC |
| 39 | MIR169n pre R | AGATCAGGCAAGTCATCCTTGGCTAC |
