# Supplementary figures and images for: Aux/IAA14 Regulates microRNA-Mediated Cold Stress Response in Arabidopsis Roots
Source: Int J Mol Sci. 2020 Nov 10;21(22):8441. doi: 10.3390/ijms21228441 (PMC7697755; doi:10.3390/ijms21228441)

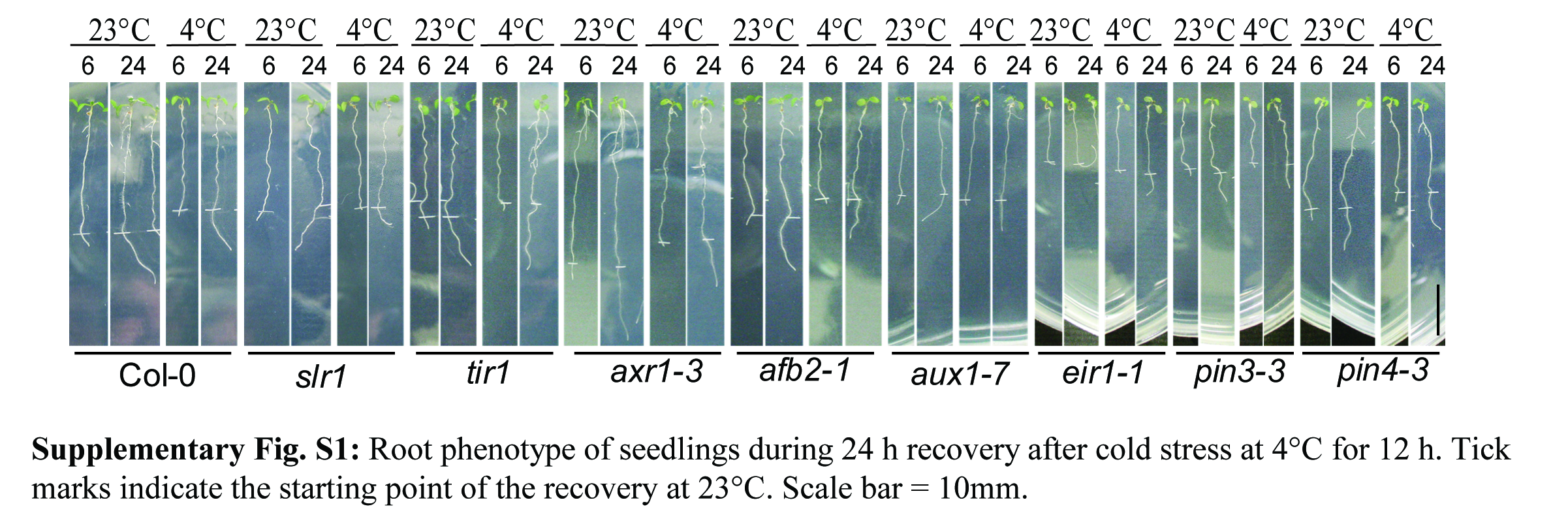

Supplement: Supplementary file 1 [file ijms-21-08441-s001.zip › Supplementary Figure S1.tif]

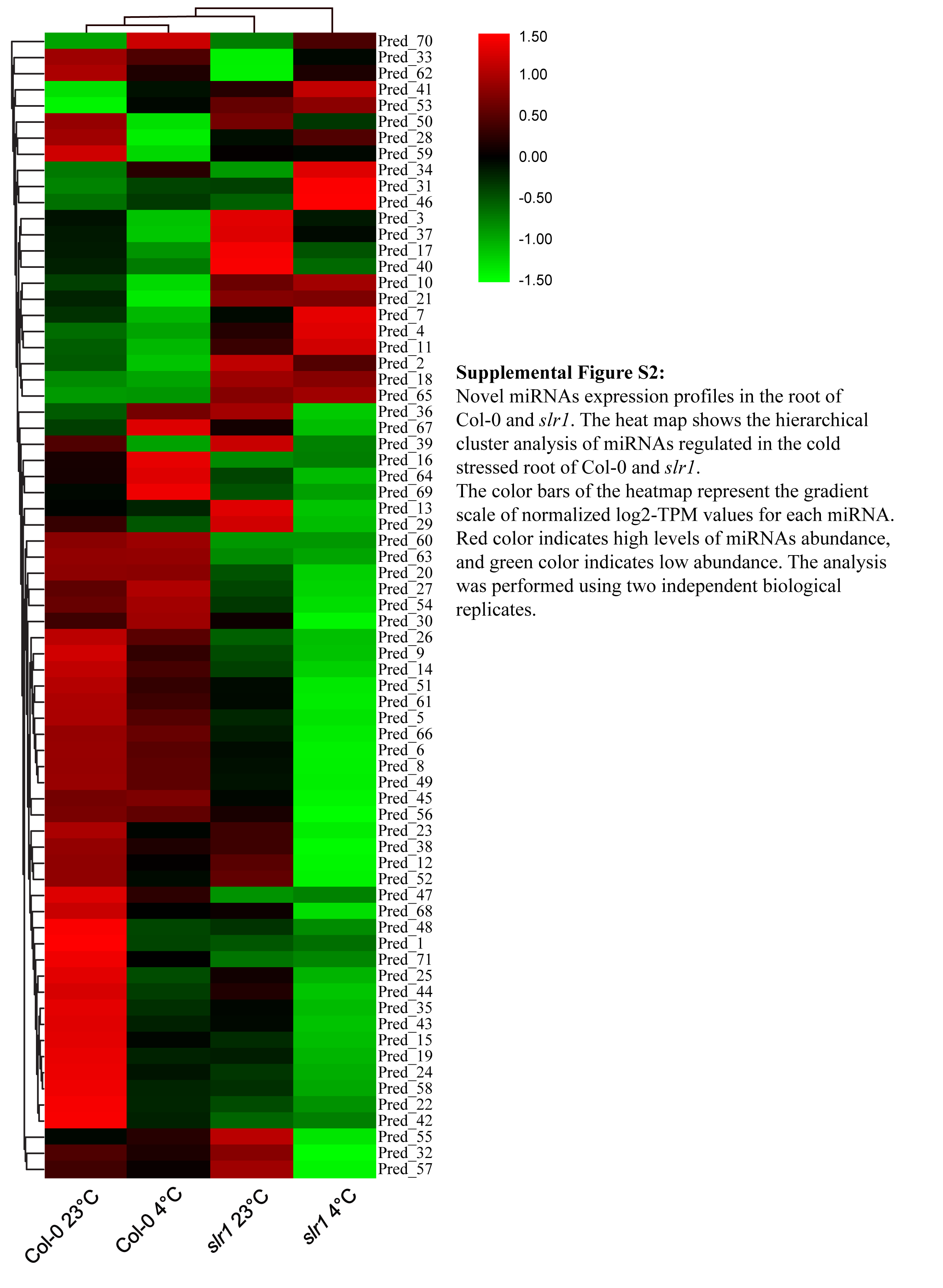

Supplement: Supplementary file 1 [file ijms-21-08441-s001.zip › Supplentary Figure S2.tif]
